# Correction: Continuous manufacturing of highly stable lead halide perovskite nanocrystals *via* a dual-reactor strategy

**DOI:** 10.1039/d6na90032a

**Published:** 2026-04-30

**Authors:** Shuang Liang, Gill M. Biesold, Mingyue Zhang, Zhitao Kang, Brent Wagner, Zhiqun Lin

**Affiliations:** a School of Materials Science and Engineering, Georgia Institute of Technology Atlanta 30332 GA USA; b School of Chemical and Biomolecular Engineering, Georgia Institute of Technology Atlanta 30332 Georgia USA; c Georgia Tech Research Institute, Georgia Institute of Technology Atlanta 30332 Georgia USA zhitao.Kang@gtri.gatech.edu; d Department of Chemical and Biomolecular Engineering, National University of Singapore Singapore 117585 Singapore z.lin@nus.edu.sg

## Abstract

Correction for ‘Continuous manufacturing of highly stable lead halide perovskite nanocrystals *via* a dual-reactor strategy’ by Shuang Liang *et al.*, *Nanoscale Adv.*, 2023, **5**, 2038–2044, https://doi.org/10.1039/D2NA00744D.

The authors regret that the name of one of the authors (Mingyue Zhang) was shown incorrectly in the original article. The corrected author list is as shown above.

The Royal Society of Chemistry apologises for these errors and any consequent inconvenience to authors and readers.

